# A case-control study of the risk factors for fistula-in-ano in infants and toddlers

**DOI:** 10.1186/s12887-024-04826-8

**Published:** 2024-05-24

**Authors:** Yanting Sun, Hongtao Liang, Shuang Hao, Lu Yin, Yibin Pan, Chen Wang, Jingen Lu

**Affiliations:** 1 Department of Anorectal Surgery, Longhua Hospital, Shanghai University of Traditional Chinese Medicine, Shanghai, China; 2 Institute of Chinese Traditional Surgery, Longhua Hospital, Shanghai University of Traditional Chinese Medicine, Shanghai, China

**Keywords:** Fistula-in-ano, Infant, Toddler, Risk factors, Case-control

## Abstract

**Background:**

Limited data are available regarding the risk factors for fistula-in-ano (FIA) in infants and toddlers, potentially affecting their daily lives.

**Objectives:**

The purpose of this study was to identify potential risk factors for FIA in infants and toddlers, in order to implement early preventive interventions, avoid disease progression, and develop therapeutic strategies.

**Design and settings:**

A retrospective case-control study was conducted, comparing 41 infants and toddlers diagnosed with FIA with 41 healthy controls, between August 2020 and December 2021.

**Independent variables:**

(a) maternal characteristics during pregnancy and delivery, (b) perinatal characteristics, dietary behaviors, and defecation-related behaviors in infants and toddlers, (c) family dietary behaviors.

**Results:**

Mothers of infants and toddlers with FIA had given birth more times in the past, while the infants and toddlers themselves had less mealtime, a higher rate of exclusive breastfeeding, frequent loose stools, and a larger proportion of used wipes, experiencing perianal skin anomalies. The logistic regression analysis revealed that there are four significant risk factors associated with the development of FIA in infants and toddlers, including the number of previous deliveries by the mother (OR 6.327), defecation frequency score (OR 5.351), stool consistency score (OR 5.017), and cleaning with wipes after defecation (OR 8.089).

**Conclusion:**

Based on our data, it appeared that FIA in infants and toddlers could be attributed to several factors. These included an increased number of previous deliveries by mothers, frequent loose stools, and repeated wipe use. To prevent the occurrence and worsening of the disease, it is important to improve the frequency and consistency of stooling and provide proper care. Further research is required to verify these findings in other clinical settings.

## Introduction

Perianal abscess (PA) and fistula-in-ano (FIA) are two relevant entities of the same disease process. FIA represents the chronic manifestation occurring simultaneously or subsequent to the formation of a PA, which is defined as an abnormal communication between perianal skin and anorectal canal [1]. Based on the classical theory of the “cryptoglandular hypothesis”, infected anal glands are considered the origination of most PAs and FIAs [2].

It has been discovered that FIA in infants and toddlers, is different from that in adults [3]. Although there is limited epidemiological data on this pediatric population, there still reveal an overwhelming male preponderance, and the majority of patients are less than 1 year of age [4]. Fistulas are generally low and simple, passing radially from the internal opening to the external opening [5].

Up to now, despite several possible reasons that have been proposed, the underlying pathogenesis of FIA in infants and toddlers remains undetermined. The causes can be congenital or acquired. Infants and toddlers with early-onset FIA were found to have an epithelial lining in the fistula tract, indicating a lesion that possibly originated from migratory cells of the urogenital sinus [6]. Another developmental defect that contributes to the occurrence of cryptitis and increases the chances of PAs and FIAs formation is an irregular thick pectinate line composed of abnormal crypts of Morgagni [7]. The predisposition to crypto glands infection might be associated with hyperfunction of anal glands induced by an excess of androgen [6, 8]. Additionally, certain underlying conditions such as anorectal malformation, inflammatory bowel disease, and immunodeficiency disorders have also been suggested as potential causes of FIA [9–11]. In infants and toddlers, FIA can sometimes be caused by injuries to the rectum and anus due to improper care [12, 13].

Although the disease is considered self-limiting for some patients, there is still great controversy over the optimal management of FIA in infants and toddlers [14]. A wide range of non-operative methods was applied to alleviate symptoms or cure the disease but success rates varied widely [15–17]. Changing feeding formula may also be a part of the treatment for patients with loose stool or diarrhea [18].

As infants and toddlers develop, they may be influenced by various factors, emphasizing the importance of considering multiple aspects when investigating factors associated with the occurrence and development of FIA in this population. Therefore, it is urgent to identify both modifiable and non-modifiable risk factors to detect and prevent diseases early, as well as prevent disease progression. This study focused on identifying distinct differences between infants and toddlers diagnosed with FIA and healthy volunteers aged 1 month to 3 years. The findings might be useful in developing new prevention strategies and offering more options for conservative treatment and postoperative care.

## Materials and methods

### Registration

The trial was registered in the Chinese Clinical Trial Registry with registration number ChiCTR2000036546 (Date of first registration: 24/8/2020). This served as the initial phase of a two-part study.

### Sample size and sample composition

Determining the sample size in this field was challenging due to the lack of sufficient historical studies. To address this, we focused on diarrhea as a risk factor for FIA in infants and toddlers. We gathered data from the pilot trial, specifically related to the typical presentation of diarrhea, which can be easily identified by caregivers [13]. We conducted a matched case-control study to compare infants and toddlers diagnosed with FIA to a control group of healthy volunteers aged 1 month to 3 years. The controls had a 0.25 probability of increased stool frequency and looser stool consistency. To detect an odds ratio of 3.00 with 90% power and a significance level of 0.05, we calculated a sample size of 41 for each group of patients. We enrolled 41 participants per group, comprising cases from Longhua Hospital affiliated to Shanghai University of Traditional Chinese Medicine, while controls were recruited from the local community between August 2020 and December 2021.

### Inclusion and exclusion criteria

We selected all infants and toddlers aged 1 month to 3 years diagnosed with FIA according to the International Classification of Diseases, 10th Revision, code K60.3 in the case group. Exclusion criteria removed infants and toddlers with other systemic diseases, such as inflammatory bowel disease, necrotizing colitis, leukemia, hidradenitis suppurativa, and congenital abnormalities of the anus and rectum. Healthy controls 1:1 matched by gender and age were collected without underlying diseases.

### Measurement

When assessing defecation frequency, it was categorized into four groups, each with a corresponding score. One point was assigned for “less than once daily,” two points for “1–2 times per day,” three points for “3–4 times per day,” and four points for “more than 5 times per day.” This assessment was conducted across three age ranges: 1–6, 7–12, and 13–36 months. To determine the overall frequency of defecation, the average score of the total points was calculated for each age range.

The stool consistency was evaluated using the Bristol Stool Form Scale (BSFS), which is a well-known evaluation scale that classifies stool form into seven different categories ranging from type 1 (Separate hard lumps, like nuts) to type 7 (Watery, no solid pieces) [19]. We assigned one point for type 1, two points for type 2, three points for type 3, and so on, and then calculated the average score of the total points of different types.

### Data collection

Our study utilized a structured electronic-based questionnaire to gather data from both groups. The questionnaire included information on demographics and baseline characteristics, information on pregnancy and delivery, and characteristics of the perinatal period and defecation-related behaviors of infants and toddlers. We additionally gathered data on the dietary behaviors of infants and toddlers, as well as those of their families. To identify risk factors, we gathered scores of defecation frequency and stool consistency based on the disease onset of cases. We received ethical approval from the Institutional Review Board at Longhua Hospital affiliated to Shanghai University of Traditional Chinese Medicine and obtained informed consent from parents or legal guardians for study participation.

### Statistical analysis

The data was inputted into Microsoft Excel and then analyzed using IBM SPSS Statistics 26.0. Descriptive statistics were computed and reported, including mean and standard deviation (SD) or median (interquartile range) for continuous variables and frequency (proportion of each group) for categorical variables. Bivariate associations connecting to the information from two groups were assessed using various tests, such as Student’s T test or Mann-Whitney U test for continuous data and Chi-square test or Fisher exact test for categorical data. Factors related to the onset of FIA in infants and toddlers were analyzed using multivariate logistic regression to determine the odds ratios (*OR*s) and 95% *CI*s. A *p*-value of less than 0.05 was considered statistically significant, while a *p*-value of less than 0.01 was considered highly statistically significant.

## Results

Demographics and baseline characteristics of cases and controls are shown in Table 1. The study included a total of 82 individuals, with 41 in each group. Each group was comprised of 39 males and 2 females, among which 10 aged 1–6 months, 5 aged 7–12 months, and the remaining 26 aged 13–36 months. The average age of the case group was 14.71 months, while that of the control group was 16.29 months. Both groups consisted mostly of ethnic Han Chinese who lived in urban areas. The parents’ mean or median age was between 30 and 32 years old, and most of them had college or above educational level. There were no significant differences found in age, gender, body weight and length, ethnicity, area of residence in infants and toddlers, or either age or education level of parents between the two groups.

**Table 1:** Demographics and baseline characteristics of cases and controls

Variables	*N*	Cases	Controls	*p*-value
Age (months) n (%)	82	14.71 (8.86)	16.29 (10.42)	0.127
1–6 months	20	10 (24.4)	10 (24.4)	1.000
7–12 months	10	5 (12.2)	5 (12.2)	
13–36 months	52	26 (63.4)	26 (63.4)	
Gender n (%)				
Male	78	39 (95.1)	39 (95.1)	1.000
Female	4	2 (4.9)	2 (4.9)	
Body weight (kg)	82	9.50 (5.90)	8.00 (7.00)	0.697
Body length (cm)	82	75.98 (13.08)	76.83 (14.08)	0.488
Ethnicity n (%)				
Ethnic Han	81	41 (100)	40 (97.6)	1.000
Minorities	1	0 (0)	1 (2.4)	
Area of residence n (%)				
Urban	74	36 (87.8)	38 (92.7)	0.712
Rural	8	5 (12.2)	3 (7.3)	
Maternal age (years)	82	30.76 (3.78)	31.68 (4.01)	0.764
Maternal education level n (%)				
Elementary school or below	1	0 (0)	1 (2.4)	0.503
Junior school	3	2 (4.9)	1 (2.4)	
High school	7	2 (4.9)	5 (12.2)	
College and above	71	37 (90.2)	34 (82.9)	
Paternal age (years)	82	31.00 (5.00)	32.00 (5.00)	0.284
Paternal education level n (%)				
Elementary school or below	2	0 (0)	2 (4.9)	0.667
Junior school	2	1 (2.4)	1 (2.4)	
High school	8	5 (12.2)	3 (7.3)	
College or above	70	35 (85.4)	35 (85.4)	

Table 2 presents the maternal characteristics during pregnancy and delivery of cases and matched controls. There appear to be differences in these characteristics between two groups. Specifically, mothers in the case group had a higher proportion of multiple previous deliveries compared to the control group. It’s noteworthy that 3 mothers in the case group had a history of 2 previous deliveries, while none in the control group did. However, there were no significant differences in other factors, including age at delivery, pregnancy-induced and pregnancy-associated disorders, body weight, drug use, and lifestyle habits during pregnancy.

**Table 2:** Comparison of maternal characteristics during pregnancy and delivery of cases and controls

Variables	*N*	Cases	Controls	*p*-value
Age at delivery	82	29.00 (5.00)	30.00 (5.00)	0.426
Number of previous deliveries n (%)				
0	59	24 (58.5)	35 (85.4)	0.012
1	20	14 (34.1)	6 (14.6)	
2	3	3 (7.3)	0 (0)	
Pregnancy-induced and pregnancy-associated disorders n (%) (e.g., gestational hypertension, gestional hyperglycemia, hyperemesis gravidarum)				
Yes	23	12 (29.3)	11 (26.8)	1.000
No	59	29 (70.7)	30 (73.2)	
Overweight during pregnancy n (%)				
Yes	16	7 (17.1)	9 (22.0)	0.781
No	66	34 (82.9)	32 (78.0)	
Drug use during pregnancy n (%)				
Yes	18	9 (22.0)	9 (22.0)	1.000
No	64	32 (78.0)	32 (78.0)	
Active smoking during pregnancy n (%)				
Yes	0	0 (0)	0 (0)	1.000
No	82	41 (100)	41 (100)	
Passive smoking during pregnancy n (%)				
Yes	12	7 (17.1)	5 (12.2)	0.756
No	70	34 (82.9)	36 (87.8)	
Alcohol consumption during pregnancy n (%)				
Yes	1	1 (2.4)	0 (0)	1.000
No	81	40 (97.6)	41 (100)	
Coffee consumption during pregnancy n (%)				
Yes	8	6 (14.6)	2 (4.9)	0.264
No	74	35 (85.4)	39 (95.1)	

Table 3 highlights the differences in perinatal characteristics, dietary behaviors, and defecation-related behaviors between the two groups of infants and toddlers. It appears that there were notable distinctions between the two groups regarding mealtime duration, primary feeding modalities, and defecation-related behaviors such as frequency, consistency, hygiene practices, and perianal skin condition. The case group exhibited a higher proportion of infants and toddlers with mealtime durations of less than 10 min and 21–30 min, as well as more breastfed infants and toddlers compared to the control group. Additionally, the case group had a significantly higher defecation frequency score and stool consistency score, and more infants and toddlers used wipes for anal cleansing compared to the control group, which preferred plain water. It’s worth to note that perianal skin conditions in healthy infants and toddlers are usually normal, with a vast majority (92.7%) falling into this category. However, in the case group, almost half of them (48.8%) are accompanied by skin flushing and erythema.

**Table 3:** Comparison of perinatal characteristics, dietary behaviors, and defecation-related behaviors of cases and controls in infants and toddlers

Variables	*N*	Cases	Controls	*p*-value
Gestational age at birth^a^ n (%)				
Preterm	2	0 (0)	2 (4.9)	0.616
Term	77	39 (95.1)	38 (92.7)	
Post term	3	2 (4.9)	1 (2.4)	
Mode of birth n (%)				
Vaginal birth	45	23 (56.1)	22 (53.7)	1.000
Cesarean Birth	37	18 (43.9)	19 (46.3)	
Number of siblings n (%)				
Singleton	80	41 (100)	39 (95.1)	0.494
Twins	2	0 (0)	2 (4.9)	
Mealtime n (%)				
< 10 min	10	7 (17.1)	3 (7.3)	0.021
11–20 min	38	18 (43.9)	20 (48.8)	
21–30 min	19	13 (31.7)	6 (14.6)	
> 30 min	15	3 (7.3)	12 (29.3)	
Primary feeding modalities n (%)				
Breastfeeding	44	30 (73.2)	14 (34.1)	< 0.001
Formula feeding	6	4 (9.8)	2 (4.9)	
Mixed feeding	10	4 (9.8)	6 (14.6)	
Complementary food	9	2 (4.9)	7 (17.1)	
Adult-like diet	13	1 (2.4)	12 (29.3)	
Main food variety n (%)				
Staple food (e.g. rice, porridege)	21	11 (26.8)	10 (24.4)	0.621
Meat and other protein food	5	4 (9.8)	1 (2.4)	
Fruits and vegetables	3	2 (4.9)	1 (2.4)	
Well-balanced diet	33	14 (34.1)	19 (46.3)	
No complementary food	20	10 (24.4)	10 (24.4)	
Taste preference n (%)				
Greasy	0	0 (0)	0 (0)	0.310
Light	20	13 (31.7)	7 (17.1)	
Moderate	42	18 (43.9)	24 (58.5)	
No complementary food	20	10 (24.2)	10 (24.4)	
Food temperature preference n (%)				
Raw and cold food	4	0 (0)	4 (9.8)	0.131
Ripe and hot food	1	0 (0)	1 (2.4)	
Moderate temperature	57	31 (75.6)	26 (63.4)	
No complementary food	20	10 (24.4)	10 (24.4)	
Defecation frequency score	82	4.00 (1.00)	2.00 (0.58)	< 0.001
Stool consistency score	82	7.00 (1.00)	4.50 (1.50)	< 0.001
Hygiene practices after defecation n (%)				
Plain water	43	15 (36.6)	28 (68.3)	0.002
Tissues	8	3 (7.3)	5 (12.2)	
Wipes	31	23 (56.1)	8 (19.5)	
Perianal skin conditions n (%)				
Normal	57	19 (46.3)	38 (92.7)	< 0.001
Dry	5	2 (4.9)	3 (7.3)	
Flushing	7	7 (17.1)	0 (0)	
Erythema	13	13 (31.7)	0 (0)	

^a^ Gestational age at birth: Preterm: < 37 weeks; Term: 37– 42 weeks; Post term: > 42 weeks

**Fig. 1:**
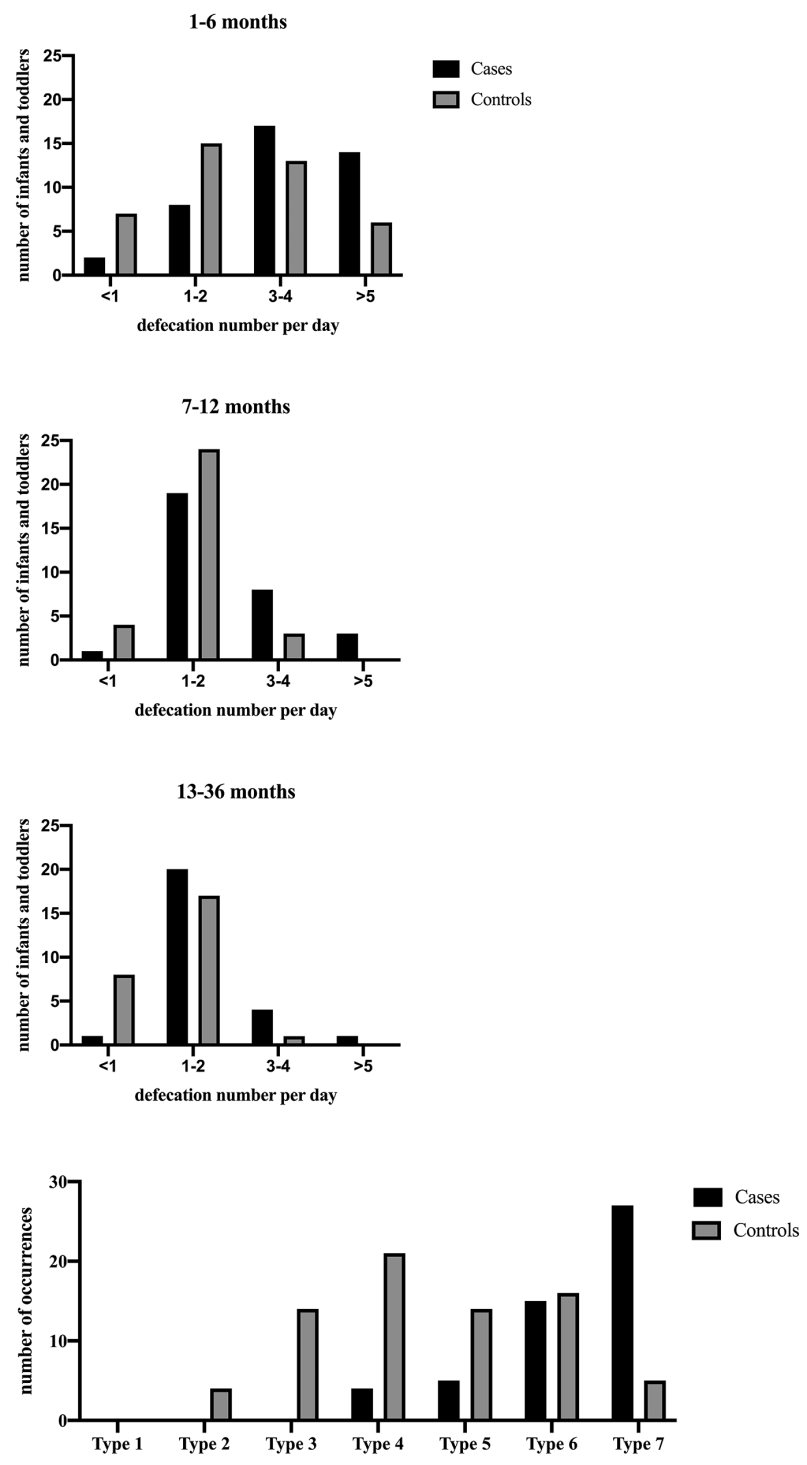
Bar graph showing the distribution of defecation frequency at different age stratification and occurrences of different stool types according to the BSFS of cases and controls

Table 4; Fig. 1 summarize the defecation frequency at different age stratification and occurrences of different stool types. It was observed that the overall defecation frequency decreased with increasing age, with the most significant differences noted between cases and controls at 1–6 months and 13–36 months. Among the control group, the most common defecation frequency was 1–2 times per day at all ages. However, infants and toddlers diagnosed with FIA were more likely to defecate over 5 times per day at 1–6 months. Furthermore, a greater proportion of infants and toddlers in the case group defecated more than 3 times per day compared to those in the control group. Healthy infants and toddlers were observed to have lower defecation frequency than those with FIA. The characteristics of stool may undergo various changes. According to the BSFS, types 1–3 were never observed in the case group, while type 7 had the highest frequency. In the control group, stools ranged from type 2 to type 7, with the highest percentage being type 4, followed by type 3 and type 6. Additionally, the occurrences of types 2–6 in healthy controls were all higher than those in cases.

**Table 4:** Comparison of defecation frequency at different age stratification and occurrences of different stool types of cases and controls

Variables	*N*	Cases	Controls	*p*-value
1–6 months defecation number per day n (%)				
<1	9	2 (4.9)	7 (17.1)	0.036
1–2	23	8 (19.5)	15 (36.6)	
3–4	30	17 (41.5)	13 (31.7)	
>5	20	14 (34.1)	6 (14.6)	
7–12 months defecation number per day n (%)				
<1	5	1 (3.2)	4 (12.9)	0.051
1–2	43	19 (61.3)	24 (77.4)	
3–4	11	8 (25.8)	3 (9.7)	
>5	3	3 (9.7)	0 (0)	
13–36 months defecation number per day n (%)				
<1	9	1 (3.8)	8 (30.8)	0.021
1–2	37	20 (76.9)	17 (65.4)	
3–4	5	4 (15.4)	1 (3.8)	
>5	1	1 (3.8)	0 (0)	
Number of occurrences of different stool types according to the BSFS^b^ n (%)				
Type 1	0	0 (0)	0 (0)	< 0.001
Type 2	4	0 (0)	4 (5.4)	
Type 3	14	0 (0)	14 (18.9)	
Type 4	25	4 (7.8)	21 (28.4)	
Type 5	19	5 (9.8)	14 (18.9)	
Type 6	31	15 (29.4)	16 (21.6)	
Type 7	32	27 (52.9)	5 (6.8)	

^b^ Bristol Stool Form Scale (BSFS): Type 1: Separate hard lumps, like nuts; Type 2: Sausage-shaped but lumpy; Type 3: Like a sausage or snake but with cracks on its surface; Type 4: Like a sausage or snake, smooth and soft; Type 5: Soft blobs with clear-cut edges; Type 6: Fluffy pieces with ragged edges, a mushy stool; Type 7: Watery, no solid pieces [19]

Based on the data collected from the two groups, it was found that there were significant differences in dietary structure and food temperature preference (Table 5). Families in the control group tended to prefer a well-balanced diet and moderate temperature, while those in the case group had varying degrees of preferences. Taste preference did not show any significant association.

**Table 5:** Comparison of family dietary behaviors of cases and controls

Variables	*N*	Cases	Controls	*p*-value
Dietary structure n (%)				
Staple food (e.g., rice, porridege)	23	14 (34.1)	9 (22.0)	0.047
Meat and other protein food	7	5 (12.2)	2 (4.9)	
Fruits and vegetables	3	3 (7.3)	0 (0)	
Well-balanced diet	49	19 (46.3)	30 (73.2)	
Taste preference n (%)				
Greasy	7	5 (12.2)	2 (4.9)	0.549
Light	22	11 (26.8)	11 (26.8)	
Moderate	53	25 (61.0)	28 (68.3)	
Food temperature preference n (%)				
Raw and cold food	3	3 (7.3)	0 (0)	0.041
Ripe and hot food	9	7 (17.1)	2 (4.9)	
Moderate temperature	70	31 (75.6)	39 (95.1)	

Further analysis was conducted using multivariate logistic regression with a backward stepwise method to identify the risk factors for FIA in infants and toddlers on the variables with significant differences. As depicted in Table 6, it was discovered that mothers in the case group had a greater number of deliveries. Additionally, infants and toddlers diagnosed with FIA were more prone to experience frequent loose stools, and the use of wipes for anal cleaning was found to increase the risk of disease compared to washing with plain water. The study thus identified the number of previous deliveries and three defecation-related behaviors as risk factors for FIA in infants and toddlers.

**Table 6:** Multivariate logistic regression analysis of risk factors for FIA in infants and toddlers

Variables	B	SE	Wald	*p*-value	OR	95% CI for Exp(B)		
						Lower	Upper	
Number of previous deliveries	1.845	0.807	5.230	0.022	6.327	1.302	30.749	
Defecation frequency score	1.677	0.631	7.070	0.008	5.351	1.554	18.424	
Stool consistency score	1.613	0.512	9.928	0.002	5.017	1.840	13.680	
Hygiene practices after defecation								
Plain water	Ref							
Tissues	-1.158	1.186	0.953	0.329	0.314	0.031	3.212	
Wipes	2.090	1.047	3.986	0.046	8.089	1.039	62.967	

## Discussion

It’s interesting to note that while there isn’t a clear understanding of the epidemiological patterns of FIA in infants and toddlers, there seems to be an increase in cases with complicated conditions. Unlike FIA in adults, the causes of the disease in infants and toddlers can be divided into two categories: congenital and acquired theories. It has previously been concluded that the abnormal crypts of Morgagni and anal glands at birth are the elicitors of cryptitis [7, 20]. While some experts suggest that frequent passage of loose or watery stools also plays a critical role in PA formation, which can then progress into FIA [5, 13]. It’s important to keep in mind that infants and toddlers may be affected by various factors from embryonic development, any of which could increase the likelihood of the disease.

This study seems to be a promising first step towards identifying the factors that contribute to FIA in infants and toddlers aged 1 month to 3 years. We adopted a comprehensive approach by collecting data from various sources, including infants and toddlers, parents, and families. It is worth noting that there were significant disparities between the mothers and infants/toddlers in the case group compared to the control group.

Several studies have established a connection between pregnancy, delivery, and the health outcomes of infants and toddlers. For instance, mothers with gestational diabetes are at an increased risk of being overweight, potentially resulting in complications such as shoulder dystocia, clavicle fractures, and brachial plexus injuries [21, 22]. However, in our study, we did not find maternal overweight during pregnancy to be one of the risk factors for the development of FIA in infants and toddlers. In China, the release of the three-child policy might lead to an increased risk of FIA in infants and toddlers with more deliveries. Repeated deliveries can also lead to childhood undernutrition, resulting in low birth weight, stunting, and anemia [23, 24]. Additionally, having more deliveries may increase the likelihood of respiratory infections in neonates [25]. It is important to note that a short birth interval may also be a significant risk factor, which was not considered in this study [26]. Further research will be needed to validate this observation.

It is quite common for healthy infants to have a relatively high defecation frequency and soft stools. Based on our research, it appears that infants and toddlers who developed FIA tend to have even more frequent bowel movements with looser stool forms than those in control groups. our study found that 34.1% of cases defecated over 5 times per day at 1–6 months, which was more than 2-fold higher compared to the controls [27]. It seemed that the primary feeding modalities have obvious effects on changes in defecation patterns, which was consistent with the present study [28]. While exclusive breastfeeding is a common and crucial feeding modality, it may not be generalizable to infants and toddlers undergoing FIA with frequent looser stools. The control of stooling by changing feeding practices had been mentioned in previous studies in treating FIA in infants, as the frequent loose or watery stools and diarrhea usually caused the disease and aggravated the condition originating from the infectious anal glands [18, 29–31]. Thus, shifts in the feeding mode of breastfeeding were occasionally required to counteract scenarios such as lactose intolerance and gastrointestinal cow’s milk allergy. Due to reduced ability to absorb lactose and allergic to cow’s milk protein, it was highly possible that infants and toddlers would suffer diarrhea, gastrointestinal reaction, and a series of clinical symptoms [32, 33]. By adjusting defecation frequency and stool consistency, the condition of patients with FIA may be improved, especially when the underlying causes are identified.

It is also important to ensure proper hygiene and care of the perianal region in infants and toddlers diagnosed with FIA. Improper care after defecation, particularly in the cleaning method of wipe use, could be an independent risk factor. It is crucial to maintain skin barrier function to avoid infections and injuries [34]. The repeated wiping resulting from frequent bowel movements seemed to increase susceptibility to damage to the rectal mucosa and perianal skin, potentially contributing to the onset of the disease [13].

In addition, there were evident differences in perianal skin condition between infants and toddlers wearing diapers and those who were not. Diaper dermatitis, a common inflammatory condition, was highest in the perianal area and had a close connection with the skin microbiome, such as *Staphylococcus aureus* [35, 36]. This specific type of bacteria has been reported as one of the infectious pathogens of PAs and FIAs in several research studies [31, 37, 38]. Family dietary behaviors could also cause apparent effects on childhood disorders, including FIAs, as dietary behaviors in infants and toddlers are often shaped by parental influence and the home food environment [39].

## Conclusion

Our research has revealed that there are various factors associated with FIA in infants and toddlers, including the number of previous deliveries by the mother, frequent loose stools, and repeated use of wipes. Primary feeding methods, perianal skin abnormalities, and unhealthy family dietary habits may also be associated with the disease. Although addressing the risk factor associated with the mother, whose mechanism related to anal fistula in infants and toddlers remains unclear, may pose challenges, other related risks can be effectively minimized by closely monitoring defecation-related behaviors and providing appropriate nutrition by caregivers. As such, it is crucial to prioritize monitoring defecation frequency and stool consistency in the growth and development of infants and toddlers diagnosed with FIA. This will aid in reducing the occurrence of diarrhea and identifying its causes. Along with this, developing appropriate feeding methods and encouraging healthy dietary habits can go a long way toward minimizing the risk of FIA and potentially reversing the disease process. It’s important to acknowledge the limitations of this study, including its retrospective design and single-center approach with a small sample size. Furthermore, as the majority of participants in both the case and control groups were recruited from Shanghai, there is a risk of selection bias that may affect the results. Therefore, extensive research with larger sample sizes across multiple centers and locations is needed to comprehensively evaluate the risk factors associated with FIA in infants and toddlers.

## Data Availability

The data generated or analyzed during this study are included in this published article and the raw datasets are available from the corresponding author on reasonable request.

## Abbreviations

FIA: fistula-in-ano
PA: perianal abscess
BSFS: Bristol Stool Form Scale
